# Supplementary material for: Pyruvate Dehydrogenase Kinase 1 inhibition mediated oxidative phosphorylation enhancement in cartilage promotes osteoarthritis progression
Source: BMC Musculoskelet Disord. 2023 Jul 20;24:597. doi: 10.1186/s12891-023-06585-6 (PMC10357736; doi:10.1186/s12891-023-06585-6)
Supplement: Supplementary file 1 — Additional file 1. [file 12891_2023_6585_MOESM1_ESM.zip › MF.Gsea.1653627316242/GOMF_POLYSACCHARIDE_BINDING.html]

Details for gene set GOMF\_POLYSACCHARIDE\_BINDING[GSEA]

|  || Dataset | 55457\_collapsed\_to\_symbols.cls.cls #OA\_versus\_normal.cls.cls #OA\_versus\_normal\_repos |
| Phenotype | cls.cls#OA\_versus\_normal\_repos |
| Upregulated in class | OA |
| GeneSet | GOMF\_POLYSACCHARIDE\_BINDING |
| Enrichment Score (ES) | 0.41322803 |
| Normalized Enrichment Score (NES) | 1.2442142 |
| Nominal p-value | 0.1682243 |
| FDR q-value | 0.47924453 |
| FWER p-Value | 1.0 |
Table: GSEA Results Summary

  

Fig 1: Enrichment plot: GOMF\_POLYSACCHARIDE\_BINDING      
 Profile of the Running ES Score & Positions of GeneSet Members on the Rank Ordered List

  

| SYMBOL | TITLE | RANK IN GENE LIST | RANK METRIC SCORE | RUNNING ES | CORE ENRICHMENT || 1 | PPP1R3C | protein phosphatase 1 regulatory subunit 3C [Source:HGNC Symbol;Acc:HGNC:9293] | 41 | 1.008 | 0.1975 | Yes |
| 2 | VTN | vitronectin [Source:HGNC Symbol;Acc:HGNC:12724] | 297 | 0.753 | 0.3275 | Yes |
| 3 | PPP1R3D | protein phosphatase 1 regulatory subunit 3D [Source:HGNC Symbol;Acc:HGNC:9294] | 708 | 0.591 | 0.4132 | Yes |
| 4 | PPP1R3A | protein phosphatase 1 regulatory subunit 3A [Source:HGNC Symbol;Acc:HGNC:9291] | 2732 | 0.277 | 0.3113 | No |
| 5 | ENPP1 | ectonucleotide pyrophosphatase/phosphodiesterase 1 [Source:HGNC Symbol;Acc:HGNC:3356] | 3392 | 0.212 | 0.3024 | No |
| 6 | AGL | "amylo-alpha-1, 6-glucosidase, 4-alpha-glucanotransferase [Source:HGNC Symbol;Acc:HGNC:321]" | 3537 | 0.199 | 0.3308 | No |
| 7 | STBD1 | starch binding domain 1 [Source:HGNC Symbol;Acc:HGNC:24854] | 6139 | -0.015 | 0.1321 | No |
| 8 | HLA-DRA | "major histocompatibility complex, class II, DR alpha [Source:HGNC Symbol;Acc:HGNC:4947]" | 6550 | -0.046 | 0.1093 | No |
| 9 | HLA-DRB1 | "major histocompatibility complex, class II, DR beta 1 [Source:HGNC Symbol;Acc:HGNC:4948]" | 7087 | -0.086 | 0.0849 | No |
| 10 | CLEC7A | C-type lectin domain containing 7A [Source:HGNC Symbol;Acc:HGNC:14558] | 7090 | -0.087 | 0.1020 | No |
| 11 | ENPP2 | ectonucleotide pyrophosphatase/phosphodiesterase 2 [Source:HGNC Symbol;Acc:HGNC:3357] | 8442 | -0.189 | 0.0349 | No |
| 12 | PTX3 | pentraxin 3 [Source:HGNC Symbol;Acc:HGNC:9692] | 9943 | -0.316 | -0.0187 | No |
| 13 | PRG4 | proteoglycan 4 [Source:HGNC Symbol;Acc:HGNC:9364] | 10035 | -0.325 | 0.0389 | No |
| 14 | FCN2 | ficolin 2 [Source:HGNC Symbol;Acc:HGNC:3624] | 10937 | -0.423 | 0.0532 | No |
| 15 | ENDOU | "endonuclease, poly(U) specific [Source:HGNC Symbol;Acc:HGNC:14369]" | 11468 | -0.498 | 0.1111 | No |
Table: GSEA details [plain text format]

  

Fig 2: GOMF\_POLYSACCHARIDE\_BINDING      
 Blue-Pink O' Gram in the Space of the Analyzed GeneSet

  

Fig 3: GOMF\_POLYSACCHARIDE\_BINDING: Random ES distribution      
 Gene set null distribution of ES for **GOMF\_POLYSACCHARIDE\_BINDING**
